# Depicting changes in land surface cover at Al-Hassa oasis of Saudi Arabia using remote sensing and GIS techniques

**DOI:** 10.1371/journal.pone.0221115

**Published:** 2019-11-14

**Authors:** Abdulrahman Mohamed Almadini, Abdalhaleem Abdalla Hassaballa

**Affiliations:** Environment & Agricultural Natural Resources Department, College of Agricultural and Food Sciences, King Faisal University, Al-Hassa, Kingdom of Saudi Arabia; Curtin University, AUSTRALIA

## Abstract

This study assessed the spatial and temporal variations of land cover in the agricultural areas of the Al-Hassa oasis, Kingdom of Saudi Arabia (KSA). Change detection technique was applied in order to classify variations among different surface cover aspects, during three successive stages between 1985 and 2017 (i.e., 1985 to 1999 (14 years), 1999 to 2013 (14 years), and 2013 to 2017 (4 years)), using two scenarios. During the first stage, significant urban sprawl (i.e., 3,200 ha) occurred on bare lands within the old oasis, while only 590 ha of the oasis’s vegetation area was occupied by urban cover. However, the final stage revealed rapid urban development (1,270 ha by 2017) within the oasis’s vegetation region, while no urban sprawl occurred on bare lands (area of 1,900 ha, same as that in 1999–2013). Vegetation cover of around 1,000 ha changed to the bare soil class, in addition to the areas that were occupied by the urban class (1,700 ha in total). The study provides quantitative information on the influence of urban development on the spatial changes in vegetation cover of the oasis, especially during recent decades.

## Introduction

Globally, approximately 1.2 million km^2^ of forests and woodlands and 5.6 million km^2^ of grassland and pasture areas have been transformed into other land use types within the last three centuries, as stated by Ramankutty and Foley [1]. Significant portions of the land surface have been transformed by humans; where 10 to 15% is currently occupied by agricultural schemes or urban-industrial areas, and 6 to 8% have been transferred into pasture lands [2]. Such alterations in land use cause significant impacts on the Earth’s climate.

Understanding how changes in land use affect land degradation requires a good understanding of the active human-environment interfaces related to land use change [3]. During the last decade, several methods to evaluate land cover changes have been proposed. These methods generate predictive models for land-use and land-cover (LULC) change.

Land cover changes can be observed by comparing sequential land-cover maps. However, assessing the fine-scale changes of land-cover types involves studying landscapes where the surface characteristics vary at seasonal and inter-annual scales in space and time [4]. Satellites provide detailed information on biophysical surface characteristics such as biomass, vegetation cover, and landscape heterogeneity. Multi-temporal analyses of these characteristics, their spatial pattern, and seasonal advancement have led to a detailed understanding of land-cover change. A wide-field of view from satellite sensors has revealed patterns of periodic disparities in land surface characteristics caused not only by change in land use but also by climatic variability.

Global urban population has been increasing more rapidly than rural populations, especially in developing countries. Built-up areas occupy up to 3% of the Earth’s land surface [5, 6]. Studies have estimated that 1 to 2 million hectares of cropland are being altered every year in developing countries in order to satisfy the demands of infrastructure, housing, industry, and others [7]. Dhaka city of Bangladesh for instance, has witnessed a substantial intensification in the built-up areas during 1975 to 2003, where built-up areas increased by 10554, having an average reaching 400 ha year-1. This tremendous expansion was attributed to a blend of different environmental, geographical and socio-economic elements [8]. City of Ajmer in India is also another example of world great urban sprawl, where expansion in the built-up areas reached 32 ha year-1 during 1977 to 2002 a stated by Jat [9].

LULC change is considered as a key factor influencing global environmental change, particularly in arid and semi-arid regions where which land and water resources are inadequate. Saudi Arabia has witnessed intense change over the last 30 years because of the economic growth due to the rise in petroleum industry and a rapid increase of urban population [10]. In a study conducted by Saudi [11], the analysis of LULC for three major cities in KSA (Riyadh, Jeddah and Makkah) revealed that urban area used to be the most altered surface cover, and most of the changed surface to urban was from bare soil during the periods from 1985 to 2014. Although a massive decrease in the agricultural lands was observed in their analysis, most of the change in agricultural lands was to bare soils due to dwindling water resources. In contrast, in the Al-Hassa oasis at the Kingdom of Saudi Arabia, a tremendous increase in population has resulted in the creation of new surface features over the last few decades [12–14], with noticeable changes in the vegetation cover. The local authorities have tried to increase agricultural efficiency by increasing areas under agriculture [15]. However, a lack of understanding, insufficient planning, and agricultural mishandling, apart from urban growth, have led to drastic changes in LULC [16]. This has caused a further degradation of the desert environment [17].

Abdelatti [14] indicated that urban growth in the Al-Hassa oasis has decreased the area under cultivation from 33% in 2009 to 25% in 2017. The authors also affirmed that such urban growth in the area without sound planning in future would have negative implications on the local environment and the social life of the residents. However, no studies have been conducted to assess the extent of green cover that has been replaced by urban sprawl in the old oasis, versus the new extensions of green cover that has been established by the government.

Remote sensing (RS) and the geographical information system (GIS) techniques have been proven to be useful tools to depict spatial and temporal changes in land cover at the Al-Hassa oasis [18–22]. This study summarizes that understanding the nature of changes in surface cover, besides quantifying the losses from cultivated lands at the Al-Hassa oasis, is of a great importance for restoration and future rehabilitation of agricultural activities.

Since the economic history of the Al-Hassa oasis is tightly associated with agricultural practices where the oasis (in its old geometry) produces a considerable share of dates in the Kingdom of Saudi Arabia (KSA), this study aimed to depict the spatial variations in the oasis’s green cover using two scenarios corresponding to urban sprawl over the past 32 years. Scenario (i) included the old oasis beside the surrounding cities, irrigation discharge lakes, and the newly embedded agricultural areas over the southern part of the oasis (i.e., the new oasis). In this scenario, the quantitative share of the new agricultural areas that extended out of the old oasis, where the new extended agricultural areas were aimed at compensating the degraded agricultural land inside the old oasis, was studied. Scenario (ii) was applied over the old oasis only in order to examine the actual change in vegetation cover (i.e., degradation) within this oasis, with respect to the other classes of surface cover throughout the estimated period (i.e., the last 30 years). In this scenario, the environmental conditions, urban sprawl, water source degradation, and the population’s social activities were assumed to have an influence on LULC changes. However, human-social impacts over the oasis were found to be the major factors.

## Material and methods

### Study area

**Fig 1:**
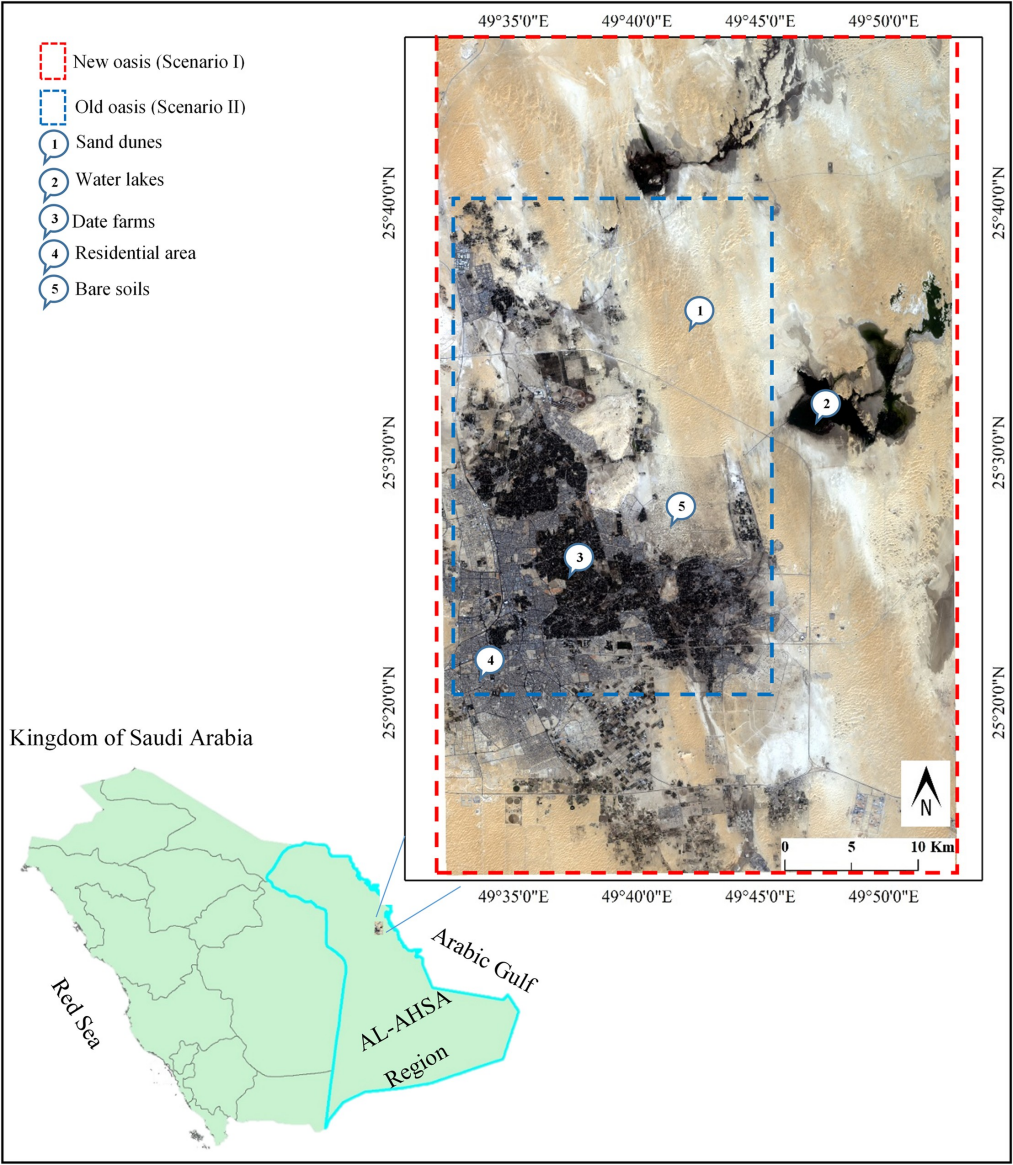
General layout and location of the study area.

This study was conducted at the Al-Hassa (i.e., Al-Ahsa) oasis, KSA. This area is considered as the largest agricultural oasis in KSA and is probably the largest irrigated oasis globally [23]. This “L” shaped oasis (Fig 1) is located at about 45 km inland from the west coast of the Arabian Gulf, 150 km south-west of Dammam city and 320 km east of Riyadh city, the capital of KSA. It is situated at altitudes raging between 160 m in the west to 130 m in the east above the mean sea level. Within the oasis, there are 10 towns and 60 villages [12].

Data from a recent study by Abdelatti [14] showed that the population of Al-Ahsa oasis increased from 445,000 in 1992 to 768,000 in 2016. According to population census 2010, the total population of the main cities (Hofuf and Mubarraz) was 660,788, which constituted 61.89% of the total province population, and 16% of the total population of the eastern region. The same was estimated to reach 768,500 by the end of 2016. The number of housing units in the province in 2017 was 149,905, representing 24.2% of the total units (618,628) in the eastern region (Statistics 2010). By 1994, around 4.4% of the buildings were made from mud and wood; in 2014 70% of the houses were converted into concrete and cement structures, 25% were made of bricks, 5% were made of stone, and there were no mud and wood houses [24].

The study area has a very gentle topography with little relief and a few surrounding ridges [25]. Active and mobile sand dunes characterize its surface as the majority of the northern, eastern, and southern boundaries of Al-Hassa are located in the Al-Jafurah desert. The sand movement/drift, estimated at 3 m^3^/m, occurs from the north-west and north [24]. The sands surrounding the oasis are mobile in nature and have for many centuries been expanding upon cultivated areas and endangering the oasis. This expansion has been tackled with measures like dune containment and tree plantations (about three million new plants).

Economically, agriculture has been the major source of livelihood for the population. Agriculture in the oasis depends on the supply of water from the numerous springs and underground sources. It has been reported by Rahman [26] that Al-Hassa is an important agricultural area for the eastern region of Saudi Arabia. The cultivated area consists of about 180 km^2^ of palm trees and oasis gardens [25]. The total area under cultivation within the oasis is approximately 80 km^2^, of which 92% is occupied by date palm [27].

Two regions of the Al-Hassa oasis were considered in this study. The first area consists of the old oasis, historically well known for its groundwater abundance [28–31], which encouraged agricultural activities in the region. This part covers an area of 20,000 ha, of which about 8,200 ha is cultivated with various fruits, vegetables, and field crops. The main crop is the date palm tree, estimated at 3 million, covering 70% or more of the total cultivated area [32, 33]. Also, the soil of this old oasis is fairly fertile and productive [34]. Thus, suitable water and soil conditions in this area encouraged the Saudi government to launch an irrigation and drainage project in 1971, which was considered as one of the most advanced water projects established in the country [35]. The project was based on a study produced by WAKUTI [28] with an aim of sustaining agricultural activities in this old oasis as well as to extend its cultivated area to encompass its total area. This cultivated area consisted of about 25,000 small farms [35].

However, in the early 70's of the last century, the Saudi government established another agricultural zone in addition to the old Al-Hassa oasis. This new area (i.e., the new oasis) is located in the Al-Ghwaibah area, south-east of the old oasis. It consisted of several farms that were distributed to the citizens. These new farms were relatively larger in area than those in the old oasis, with an area of 5 ha or more each. The soil of this new area was affected by high salinity and calcium carbonate contents but was low in organic matter [36]. This area also lacked drainage systems, in contrast to the old oasis where a network of drainage system was available that culminated at two evaporation lakes forming eminent water bodies. The first lake (Al-Uyon) is located in the north east, while the second (Al-Asfer) is located to the east of the old oasis. The Al-Hassa oasis is classified as having an under hyper-arid climate dominated by severe hot and dry conditions, causing the pan-evaporation (2000 mm yr^-1^) to significantly exceed annual rainfall (80–90 mm), with average temperatures fluctuating between 38°C in summer to 15°C in winter [37].

### Data collection and processing

As the oasis has witnessed a drastic change in the environmental, social, ecological, and demographical levels throughout the last 30 years, an area of about 1,500 km^2^ over the Al-Hassa oasis, including its cities and surrounding suburbs, was masked and selected for change detection analysis, as shown in (Fig 1).

Four cloud-free satellite images from the Landsat series were acquired for the assessment period (1985 to 2017), with a spatial resolution of 30 m (Table 1). These spatial data sets were acquired from the archives of the USGS Earth Explorer website (http://earthexplorer.usgs.gov/), and calibrated using the data-specific utilities of ENVI (Ver. 5.3) software, where the image's digital number was transformed into spectral radiance (Lλ). Subsequently, reflectance images were generated from the radiance pixels. Atmospheric correction tools such as dark object removal, haze removal, and cloud masking were used to correct the sensor radiance for atmospheric effects using Fast Line-of-sight Atmosphere Analysis of Spectral Hypercubes (FLAASH). FLAASH is a physics-based method for atmospheric correction that employs temporal and spatial metadata to develop a radiative transfer model using MODTRAN 4 [38]. Image enhancement and linear histogram stretching were also performed. Exo-atmospheric reflectance (reflectance above the atmosphere) was applied using published post-launch gain value in ENVI, which is a value that is multiplied by the pixel value to scale it into physically meaningful units of radiance: Radiance = DN * gain + offset, where offset was used in the context of remote sensing. The Lλ was calculated using the calibration coefficients from the metadata of the acquired image. Hence, reflectance value of images were determined from the obtained radiance values.

**Table 1:** Acquired satellite images.

Sensor	Sensor ID	Date of acquisition	Path/Row	Spatial resolution
Landsat-5	TM	Jan. 06, 1985	164/042	30 (m)
Landsat-7	ETM	Nov. 05, 1999	164/042	30 (m)
Landsat-8	OLI	May 11, 2013	164/042	30 (m)
Landsat-8	OLI	Dec. 16, 2017	164/042	30 (m)

In the pre-processing stage, image enhancement was conducted in order to improve the contrast between features in the images and to improve the visual interpretation of surface features. This involved manipulating the range of input digital values to create a new range of output values. For any possible atmospheric attenuation, FLAASH model was applied, which was found to be capable of producing highly precise surface reflectance values, although it required significant user inputs [38].

In scenario (i), a subset of 35 km by 50 km was masked over the entire Al-Hassa area. Scenario (ii) on the other hand, was represented in a subset mask of 10 km by 20 km; where the area was designated to be confined around the old oasis boundaries. Hence, only four surface cover classes occupied the area, namely: the vegetation cover (represented by the date palm trees), the urban area, the bare lands, and the sand dunes.

### Image classification

The acquired images were processed using supervised classification and five basic class types in scenario (i) were determined; namely: vegetation cover, urban area, bare lands, sand dunes, and water bodies. Water bodies as a class was not included in scenario (ii) as no water body was located within the borders of this scenario. Training and testing sites were selected visually from the images, assisted with a high spatial resolution basemap (0.6 m) provided by the ArcGIS (10.5) software. An image processing software system (ENVI 5.3) was then utilized to produce a statistical depiction of the reflectance for every information class. This phase is usually known as "signature analysis" and characterized the mean or the range of reflectance on each band and variances and covariance of overall bands. Upon achieving the statistical characterization for every class information, images were classified by analyzing the reflectance for every pixel and deciding on the signatures that were similar to the most frequent object. The "maximum likelihood" classifier was applied in this study. This is a supervised classification technique derived from the Bayes theorem, which employs the discriminant function to assign each pixel to the class with the highest likelihood [39]. This classifier is considered to provide better outcomes when compared to other types such as the parallelepiped and the minimum distance classification machines. However, it is significantly slower due to extra calculations involved in the process.

Training and testing sites of the five classes (developed in the classification scheme) were digitized as areas of interest (AOI), producing the five identified regions based on their spectral signatures. Hence, the assessment of classification accuracy was done using the testing points, which were extracted randomly (using randomizer machine in ENVI 5.3) from all the points, with percentages of 40% and 60% for the testing and training points, respectively.

### Accuracy assessment

A confusion matrix (also known as error matrix) is typically used as a numerical technique for portraying the accuracy of the classified image. It is set in a tabular form that illustrates correspondence between the result of the classification process and a reference image. In order to generate the confusion matrix, ground truth data, such as field observations documented with a GPS, map information, or a digitized image, are needed.

The kappa coefficient is an important measure of matching the classification. When the kappa coefficient value is 0, it indicates no similarity between the classified image and the reference image. If the value equals to 1, the classified image and the reference image are completely identical. Thus, a higher kappa coefficient indicates an accurate classification [40].

In order to assess the accuracy of classification, classification errors were identified. Hence, omission and commission errors are assessed. For every class, errors of commission happen once a classification process allocates pixels to a specific class that actually do not belong to it. The total of commission errors is then defined by an indicator known as producer’s accuracy, which is the total of correctly identified pixels divided by the total of the reference image pixels. Omission errors, on the other hand, arise once pixels that actually belong to one class, are classified as some other class. User’s accuracy is the index that characterizes the sum of omission errors in which the total number of the correctly identified pixels of a class are divided by the total pixels of that class [40].

### Change detection

In the post-classification process, image differencing technique was applied for each of the two images. This technique uses change detection statistics to provide a detailed tabulation of changes between the two classified images. The statistical report includes a class-for-class image difference. The analysis focuses primarily on the initial state classification changes. Hence, for each initial state class, the analysis identifies the classes into which the corresponding pixels changed in the final state image [41]. ENVI 5.3 can report changes as pixel counts, percentages, and areas. In addition, a special type of mask image (classification masks) can also be produced so as to provide a spatial context for the tabular report.

**Fig 2:**
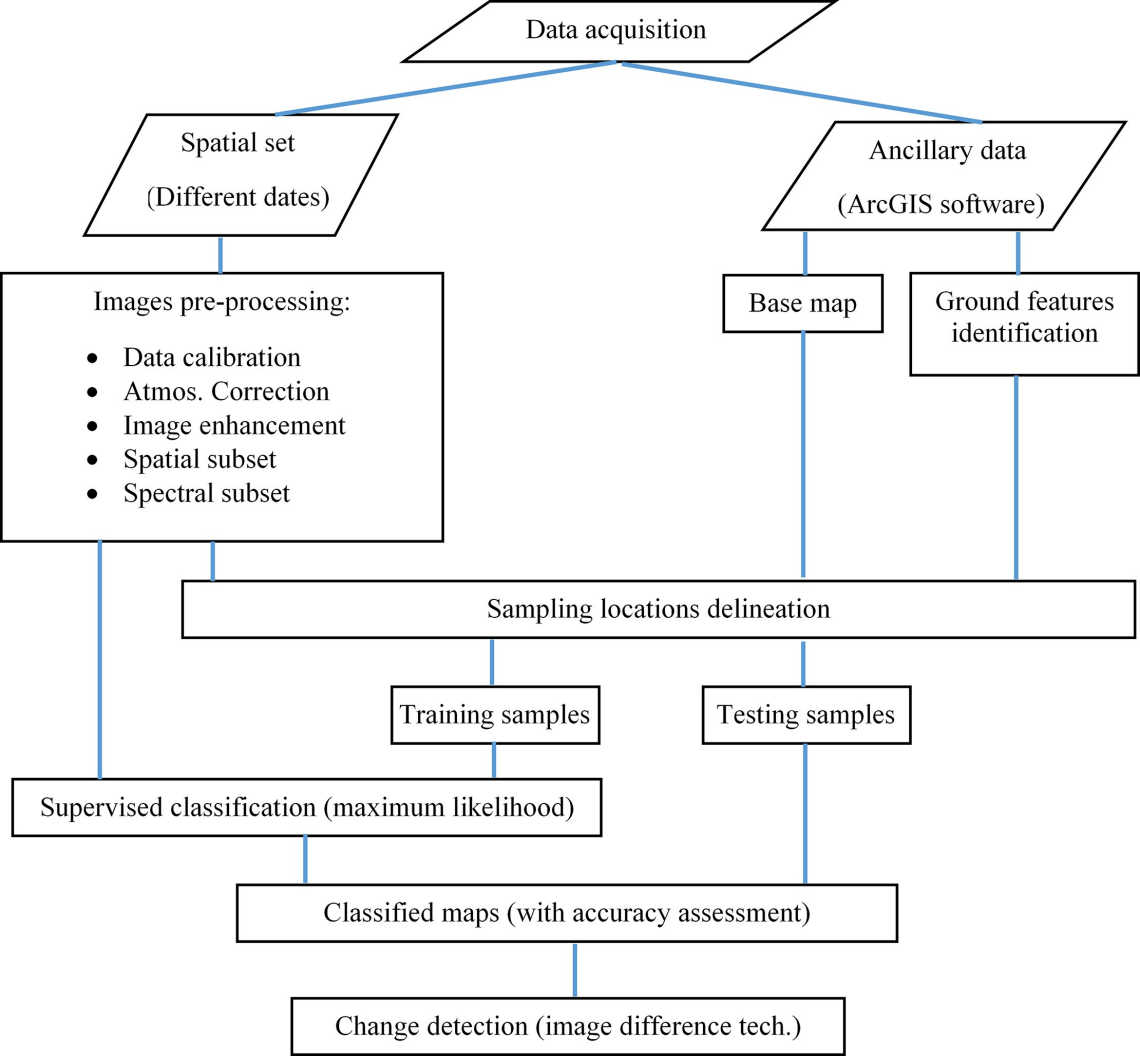
Procedure for change detection.

The class masks are classification images with class colors matching the final state image, making it easy to identify not only where the changes have occurred but also the class into which the pixels have changed (Assisting catalog of ENVI 5.3). The flow chart shown below (Fig 2) represents the procedure followed for satellite (Landsat series) image acquisition, preprocessing, classification, and the application of change detection techniques. Samples for training and testing were masked using a high spatial resolution base map (0.6 m) provided with the software packages within ArcGIS 10.5. A verification of the distinguished ground locations was achieved with the local knowledge of the authors in visually interpreting features on the base map as well as the processed images.

## Results

**Fig 3:**
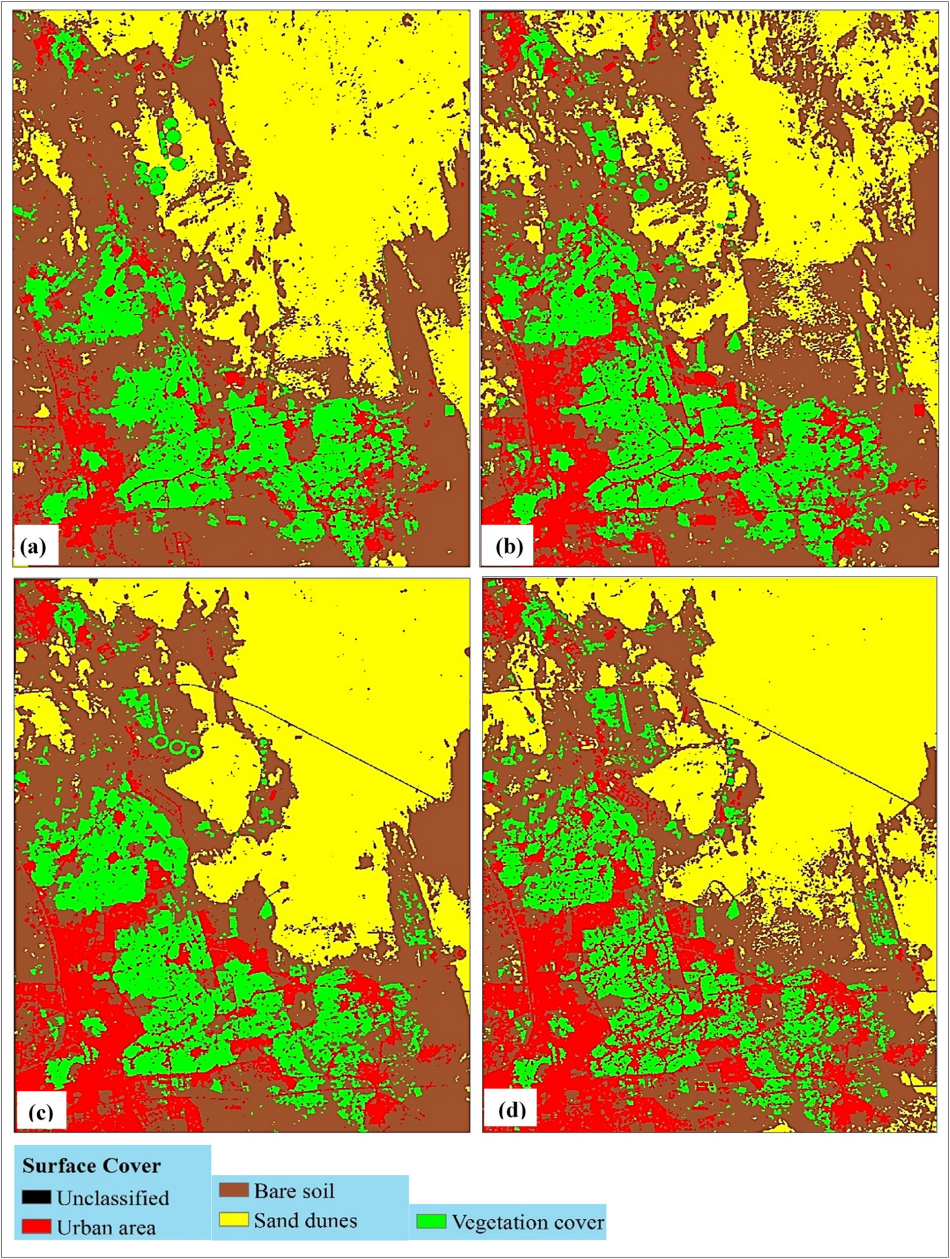
Classified maps of the study area based on scenario (i) for the years of 1985(a), 1999(b), 2013(c), and 2017(d).

**Fig 4:**
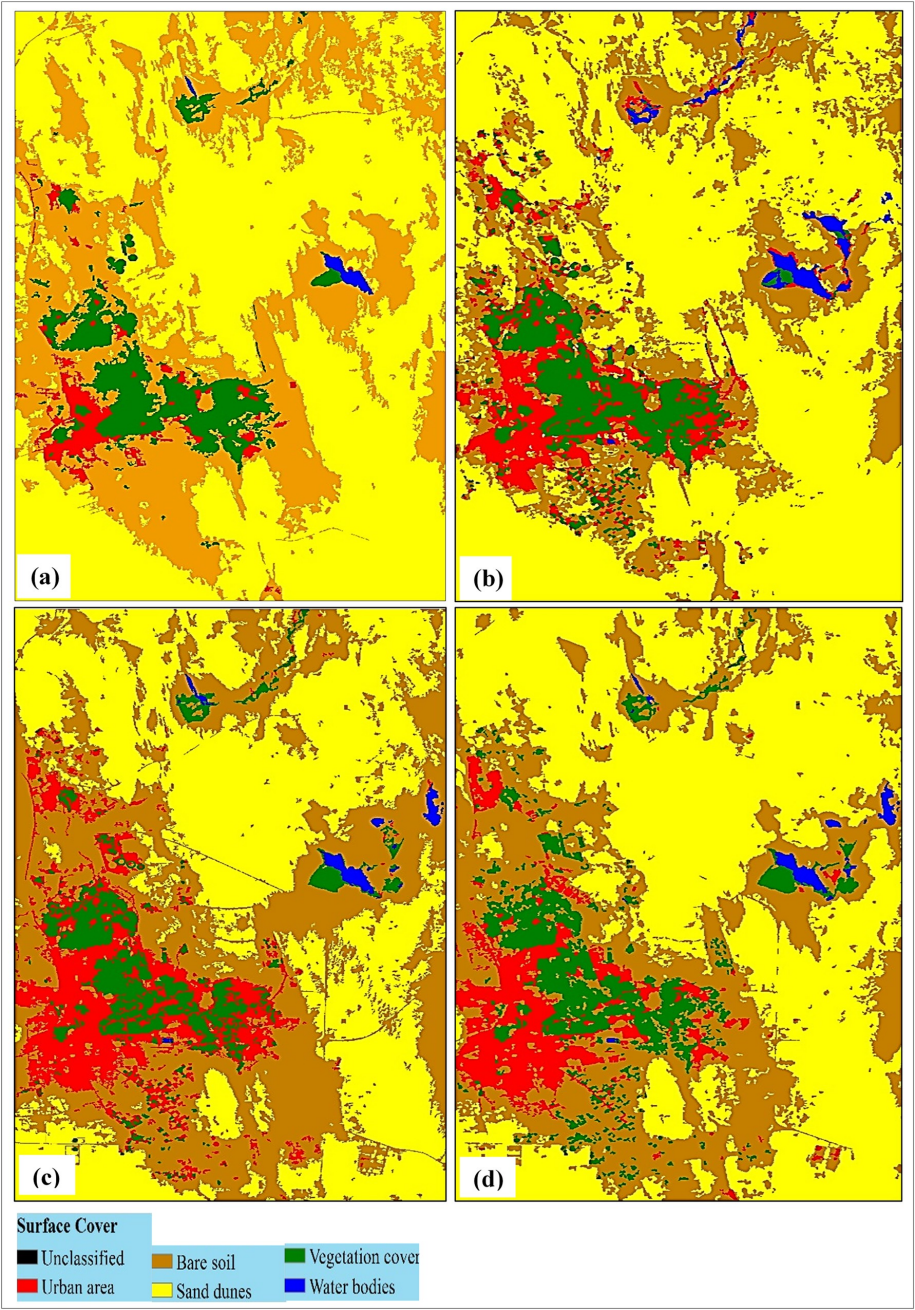
Classified maps of the study area based on scenario (ii) for the years of 1985(a), 1999(b), 2013(c), and 2017(d).

Figs 3 and 4 show the resultant classification maps of the study area for the years of (a) 1985, (b) 1999, (c) 2013, and (d) 2017 obtained from scenarios (i) and (ii), respectively. A clear spatial variability in vegetation cover class was observed in scenario (i) due to the compensation plans.

### Confusion matrix

Table 2 shows the resultant confusion matrix obtained using the pre-delineated ground truth region of interest (ROI) tools, in order to compute the classification accuracy metrics. ENVI 5.3 was utilized for achieving the same, where table columns represent the percentages of true (ground truth values) classes, whereas rows signify the percent of the classifier's predictions. This analysis was done for scenario (ii) only because the dynamic nature in surface cover at the old oasis that was observed in the form of urban development made it the focal point of the study.

**Table 2:** Confusion matrix for images classified for scenario (ii).

Predictor	Class for 1985	Ground Truth (%)				Commission	Omission	Producer	User
		Urban Area	Vegetation Cover	Bare Soil	Sand Dunes	(%)	(%)	Accuracy (%)	Accuracy (%)
	Urban Area	93.65	0.00	2.38	0.00	2.48	6.35	93.65	97.52
	Vegetation Cover	0.00	100.00	0.00	0.00	0.00	0.00	100.00	100.00
	Bare Soil	1.59	0.00	97.62	0.79	2.38	2.38	97.62	97.62
	Sand Dunes	4.76	0.00	0.00	99.21	4.58	0.79	99.21	95.42
	Total	100.00	100.00	100.00	100.00				
	Overall Accuracy (%) = (492/504) 97.62%								
	Kappa Coefficient = 0.9683								
Predictor	Class for 1999	Ground Truth (%)				Commission	Omission	Producer	User
		Urban Area	Vegetation Cover	Bare Soil	Sand Dunes	(%)	(%)	Accuracy (%)	Accuracy (%)
	Urban Area	100.00	0.00	0.00	0.00	0.00	0.00	100.00	100.00
	Vegetation Cover	0.00	100.00	0.00	0.00	0.00	0.00	100.00	100.00
	Bare Soil	0.00	0.00	100.00	0.00	0.00	0.00	100.00	100.00
	Sand Dunes	0.00	0.00	0.00	100.00	0.00	0.00	100.00	100.00
	Total	100.00	100.00	100.00	100.00				
	Overall Accuracy (%) = (928/928) 100.00%								
	Kappa Coefficient = 1.00								
Predictor	Class for 2013	Ground Truth (%)				Commission	Omission	Producer	User
		Urban Area	Vegetation Cover	Bare Soil	Sand Dunes	(%)	(%)	Accuracy (%)	Accuracy (%)
	Urban Area	94.63	0.00	3.31	0.00	3.38	5.37	94.63	96.62
	Vegetation Cover	0.00	100.00	0.00	0.00	0.00	0.00	100.00	100.00
	Bare Soil	5.37	0.00	96.69	0.00	5.26	3.31	96.69	94.74
	Sand Dunes	0.00	0.00	0.00	100.00	0.00	0.00	100.00	100.00
	Total	100.00	100.00	100.00	100.00				
	Overall Accuracy (%) = (947/968) 97.83%								
	Kappa Coefficient = 0.9711								
Predictor	Class for 2017	Ground Truth (%)				Commission	Omission	Producer	User
		Urban Area	Vegetation Cover	Bare Soil	Sand Dunes	(%)	(%)	Accuracy (%)	Accuracy (%)
	Urban Area	97.71	0.00	1.83	0.00	1.84	2.29	97.71	98.16
	Vegetation Cover	0.00	100.00	0.00	0.00	0.00	0.00	100.00	100.00
	Bare Soil	2.29	0.00	97.25	0.00	2.30	2.75	97.25	97.70
	Sand Dunes	0.00	0.00	0.92	100.00	0.91	0.00	100.00	99.09
	Total	100.00	100.00	100.00	100.00				
	Overall Accuracy (%) = (861/872) 98.73%								
	Kappa Coefficient = 0.9832								

The overall accuracies of surface cover were found to be 97.6%, 100%, 97.8%, and 98.7% for the urban area, vegetation cover, bare soil, and sand dune classes, respectively. This indicates a high similarity between the classifiers and the predictors, especially for the year 1999. During the process of error evaluation, > 94% for both producer and user accuracies were achieved with a kappa coefficient of more than 0.96 for all classified images.

### Classification statistics

The summary statistics of the acquired areas of each surface cover class (ha) under scenarios (i) and (ii) throughout the analyzed periods (i.e., 1985, 1999, 2013, and 2017) is presented in Tables 3 and 4, respectively. The range value (ha) for each class was also produced as the difference between the early state (1985) and the later state (2017), in order to reveal the final state for each class. Therefore, the resulting ranges showed that the urban area class produced the highest change in surface cover (347.29%). Scenario (i) shows that the sand dunes class was the biggest and the most dominant among the others (Table 3). Its areas ranged from 125,997.12 to 114,475.68 ha from 1985 to 2017, with a noticeable fluctuation during the analyzed period, though with no apparent trend. Regardless of such fluctuation during the whole period, the classified maps showed that the area of this class declined by nearly 11,521.38 ha (i.e., - 9.14%).

**Table 3:** Summary statistics for the acquired areas of each surface cover class (ha) by scenario (i).

Image Date	Areas (ha) of the surface cover classes (Class change %)				
	Vegetation	Urban	bare lands	Sand dunes	Water bodies
**1985**	13,105.44 (6.07)	4,597.02 (2.13)	71,642.88 (33.18)	125,997.12 (58.35)	601.38 (0.28)
**1999**	13,510.08 (6.41)	10,733.22 (5.09)	52,221.00 (24.78)	132,792.00 (63.00)	1,508.94 (0.72)
**2013**	12,455.28 (5.94)	14,148.54 (6.75)	85,472.91 (40.79)	96,197.94 (45.91)	1,255.50 (0.60)
**2017**	15,390.54 (6.92)	20,562.21 (9.25)	70,693.47 (31.79)	114,475.68 (51.48)	1,235.61 (0.56)
**Range**	+ 2,285.10	+ 15,965.19	- 949.41	- 11,521.44	+ 634.23
**Gain/Loss**	Gain	Gain	Loss	Loss	Gain
Total change (%)*	+ 17.44	+ 347.29	- 1.33	- 9.14	+ 105.46

* Statistical change given as a percentage of the early stage (1985).

**Table 4:** Summary statistics for the acquired areas of each surface cover class (ha) by scenario (ii).

Image Date	Area (ha) of the surface cover classes (Class change %)			
	Vegetation	Urban	bare lands	Sand dunes
**1985**	9,095.40 (13.81)	4,427.37 (6.72)	29,015.91 (44.07)	23,298.57 (35.39)
**1999**	10,644.93 (16.17)	7,524.63 (11.43)	30,208.32 (45.88)	17,459.37 (26.52)
**2013**	9,795.42 (14.88)	8,353.08 (12.69)	24,837.66 (37.73)	22,851.09 (34.71)
**2017**	8,472.42 (12.87)	10,645.20 (16.17)	23,028.48 (34.98)	23,691.15 (35.98)
**Range**	- 622.98	+ 6,217.83	- 5,987.43	+ 392.58
**Gain/Loss**	Loss	Gain	Loss	Gain
Total change (%)*	- 6.85	+ 140.44	- 20.63	+ 1.68

* Statistical change given a percentage of the range to the early stage (1985).

In addition, scenario (i) shows that the area of bare land class was the second highest, followed by the vegetation class (Table 3). The area of vegetation class was estimated at 13,105.44 ha in 1985 and reached 15,390.54 ha in 2017; denoting a gain of 2,285.10 ha upon other classes. The urban area that was estimated at 4,597.02 ha in 1985 reached 20,562.21 ha by 2017 due to urban sprawl. The urban area expanded nearly 16,000 ha over the other classes by the end of the analyzed period.

The class of water bodies, represented by agricultural drainage water evaporation lakes, occupied only a small portion of the surface cover of the new oasis (Table 3). However, it exhibited a noticeable increase (~100%) in area, rising from 601.38 ha to 1,235.61 ha between the years of 1985 and 2017. It reached its maximum value in 1999 (1,508.94 ha). Finally, it is worth to mention from scenario (i) of the new oasis that the areas of the sand dunes and bare lands classes were the most dominant in terms of area, which reflects the area geographical identity, where the area of these two classes represented together (i.e., 87%) of the total area (Table 3).

Though scenario (ii) was applied in order to examine the actual change in vegetation cover within the old oasis (only) with respect to the other classes of surface cover, that both sand dunes and bare soil classes masked most of the old oasis surface cover (73.81%) (Table 4).

The area of vegetation cover in scenario (ii) was third largest in the category among other classes (Table 4). It showed a loss in its area from 9,095 ha in 1985 to 8,472 ha in 2017 (- 6.85%). This trend is opposite to that of scenario (i), where there was an increase of + 17.44% over the same period (Table 3). This implies that the actual loss in the vegetation class occurred in the old oasis of Al-Hassa. The loss in this class in scenario (ii) however corresponded with a huge gain in the area of the urban class that increased from 4,427 ha in 1985 to 10,654 ha in 2017, gaining about 6,218 ha (+ 136.39%) (Table 4). The urban class showed an increasing trend throughout the study period (1985 to 2017) in both the scenarios. A major part of this sprawl has occurred in the new oasis, as was verified from Figs 2 and 3. This increase reflects the continuous increase in population and their endeavor to settle within the green spots, alongside some other social and economic considerations [12, 14, 42].

**Fig 5:**
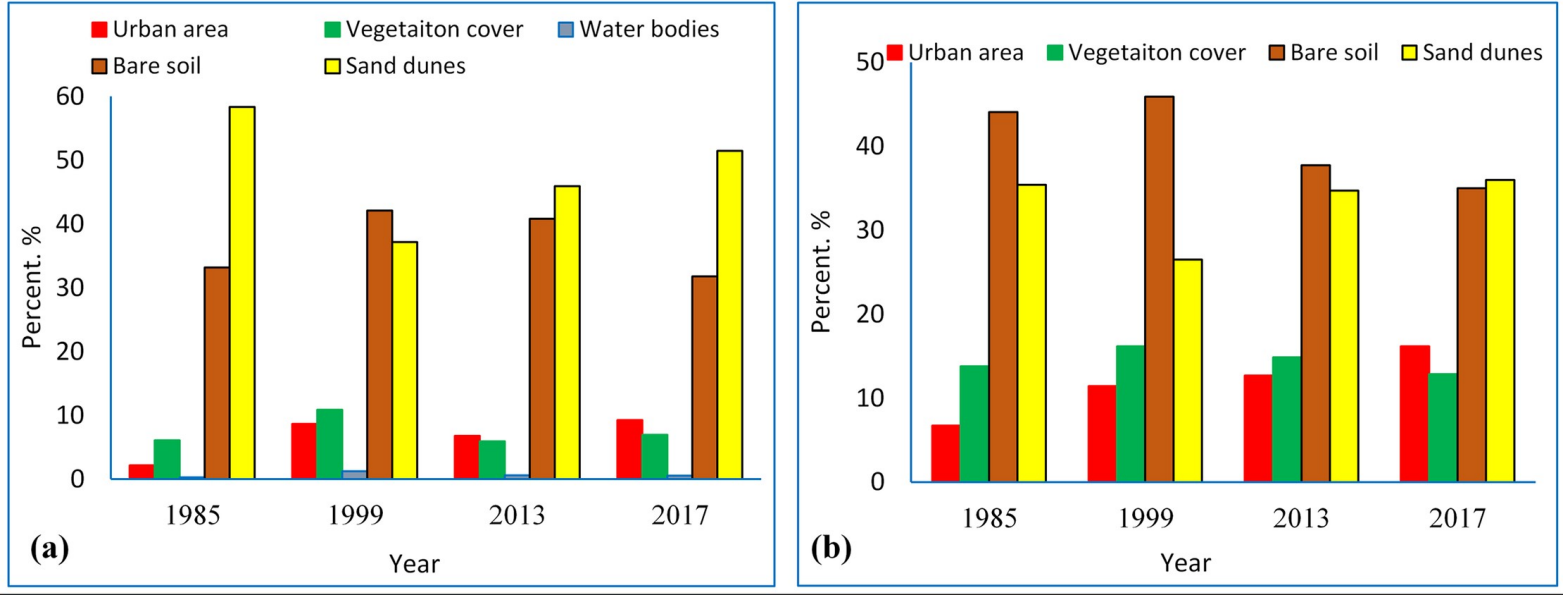
The percentages of spatial change in surface cover classes for (a) scenario (i) and (b) scenario (ii).

The percentage spatial change in each class is presented in Fig 5A for scenario (i) and Fig 5B for scenario (ii). The primary Y-axis (on the left hand) represents the percentage scale for the vegetation, urban area, and the water bodies classes, while the secondary Y-axis (on the right hand) shows the percentage scale for the bare land and sand dunes classes. From both the figures, it can be observed that there were drastic changes in the urban area class throughout the yearly time series, while the vegetation cover class showed some increase during 1985 to 1999 and then started decreasing, particularly in the old oasis. Also, an acute decrease in the area of sand dunes can be noticed during the year 1999, which could be assigned to land conservation practices that was applied to the north of the old oasis [23, 25, 43].

Referring to the percentages shown in Fig 5B, it can be concluded that the urban area class had a drastic change, as 16% of the entire oasis land was occupied by this class by 2017 as compared to 6% in 1985. Also, a slow expansion occurred in vegetation cover during 1985 to 1999 in scenario (i). Yet, a continuous decrease in the vegetation cover area, estimated as 4% of the total oasis area, was noticed during 1999 to 2017, with a simultaneous expansion in the urban area within the boundary limits of the old oasis (Fig 3). However, a major portion of the urban area expansion could not be includes as relative changes as this area was allocated outside the designated oasis boundary limits. Thus, the change detection technique was used to quantitatively assess factors affecting vegetation area losses.

### Change detection

**Fig 6:**
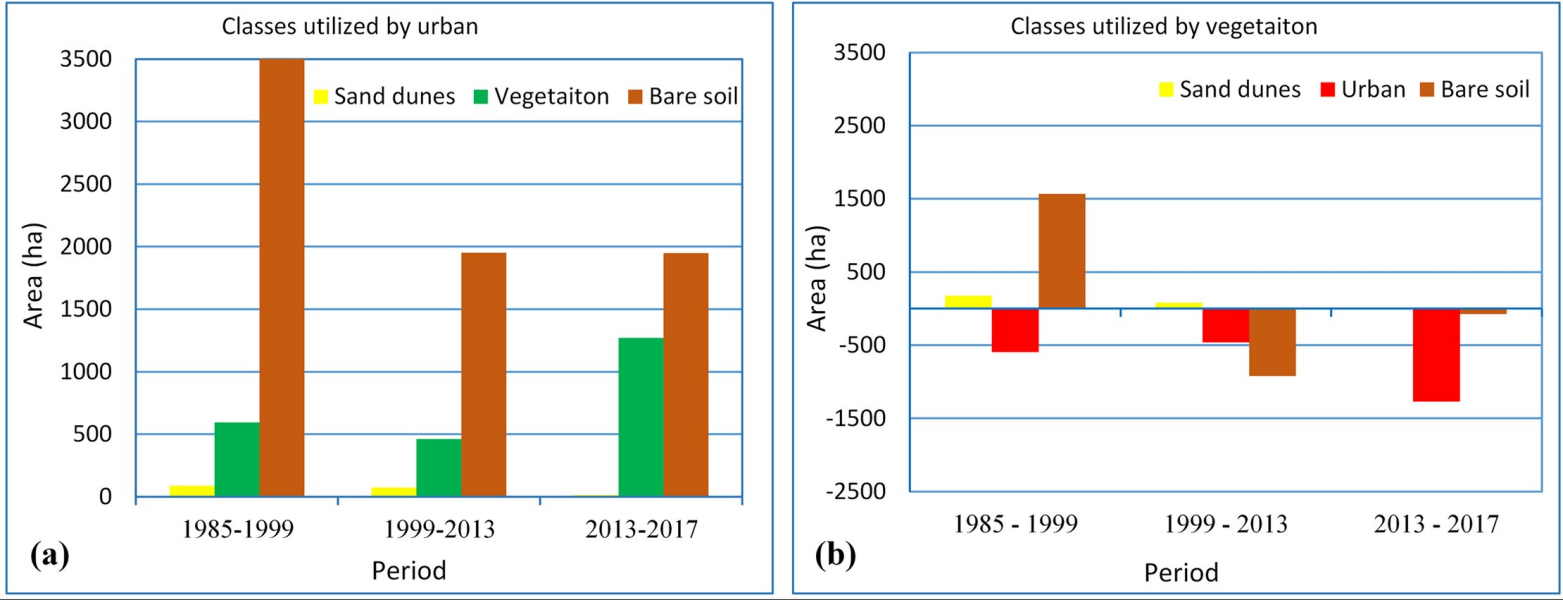
Resultant changes produced by images differencing, where (a) shows the urban sprawl over other classes and (b) shows the changes in vegetation cover corresponding to other classes.

In order to assess the quantitative gain/loss of area in the old oasis, the study area from scenario (ii) was used for detecting the changes in vegetation cover. Hence, the study focused on detecting changes in urban area and vegetation cover classes using image differencing. The change was computed by applying a segmental subtraction for each of two classified images representing specific dates. Three successive periods were used for the change analysis: 1985 to 1999 (14 years), 1999 to 2013 (14 years), and 2013 to 2017 (4 years). The values of gain/loss in area (in ha) are given for the urban area (Fig 6A) and the vegetation cover (Fig 6B). From Fig 6A, it can be seen that during the first period (1985 to 1999), almost all urban sprawl occurred upon the bare land class that used to be vacant within the oasis, and was estimated to be 3,200 ha; whilst only 590 ha of the oasis vegetation area was occupied by the urban class, in addition to few hectares (87 ha) of sand dunes that were converted into urban areas. The second period (1999 to 2013) showed a gradual decrease in urban sprawl over bare lands, where 1,900 ha of bare lands were occupied by urban areas. However, the final period (2013 to 2017) witnessed different values of change where a quick pace of urban (1,270 ha) development took place over the oasis's vegetation area, while no increase in urban sprawl took place over bare lands (1,900 ha, same as 1999 to 2013).

As shown in Fig 6B, analysis of oasis vegetation cover showed that a significant increase in vegetation cover occurred over the bare lands during 1985 to 1999, estimated at 1,560 ha as compared to 176 ha over sandy soils. However, no noticeable increase in vegetation cover occurred during 1999 to 2017, except for few hectares (80 ha) over the sand dunes. Instead, vegetation cover class lost an area of around 1,000 ha to the bare soil class in total, in addition to the areas that were occupied by the urban class (1,700 ha in total), as indicated in Fig 6A.

## Discussion

In spite of the spatial/temporal variation in bare land and sand dunes classes, the analysis showed that the bare land and sand dunes were the dominant land cover classes in both scenarios, the old one (40.66% and 33.15%, respectively) and the new one (32.42% and 54.35%, respectively). This concurs with the geographic location of the oasis, where it is surrounded by deserts that feature shifting sand dunes [23], presenting a long lasting ingress of sand on cultivated fields [13, 31, 43], despite the efforts devoted by the Saudi government to control the movement of these dunes toward the oasis [43]. The dominance of the sand dunes class in the Al-Hassa oasis was previously reported by Salih [25], who also indicated that sand dunes is the dominant land cover in the oasis, occupying up to 70% of the area, as compared to other classes including water bodies, Sabakha, bare soil, urban, and agriculture. This is also supported with other studies that indicated that the Al-Hassa oasis is located in a desert area featuring shifting sand dunes [23, 25, 43].

Furthermore, the results showed that a big portion of urban sprawl (3,200 ha) during the first stage (1985–1999) occurred over the bare land class that used to be vacant within the old oasis, while only 590 ha of the oasis's vegetation area was occupied by the urban class. This was in line with findings of a research achieved by (Saudi)[11] over three example locations in the Kingdom of Saudi Arabia (Riyadh, Jeddah and Makkah). The final period, however, witnessed a different direction of changes, where 1,270 ha of the urban class took over the oasis's vegetation area with no urban sprawl in upon bare soils (1,900 ha, same as 1999–2013), unlike most of the achieved researches in the surroundings. This could be attributed to the social activities, which highlight the cultural landscape with components of natural heritage in the agricultural practices. These new surface features found in the form of urban components, caused by the tremendous increase in population (accompanied by lack of bare lands within the old oasis) during the last few decades [12–14], occurred at the expense of the vegetation cover. This finding agrees with a regional LULC analysis conducted by (Moroco)[44], which stated that because of anthropogenic activities like urban sprawl, overgrazing, and degradation of forest sector in Béni‑Mellal District (Morocco) during 2002 to 2016, agriculture in addition to forest surface covers decreased by 40.64% and 53.85%, respectively. On contrast, there was some expansion in the green area during 1985 to 1999, which might be due to the inclusion of cultivated projects located in the northern part of the old oasis. Abdelatti [14] emphasized the threat of urban growth in the Al-Hassa oasis to the local environment. The authors also discussed that such urban growth in the area without sound planning in future would impose negative implications on the local environment and social life.

Urbanization has indirectly affected agriculture as a consequence of development in the KSA. Exploitation of oil has resulted in an increase in immigration to urban areas, principally in the Eastern Province. Although the oil business has created direct employment opportunities at the oilfields, unintended employment opportunities also came up in cities all over the country. The study by Al Jabr [43] showed that the high percentage of urban development was due to the rapid rise in immigration to the urban areas, in addition to the rise in population of their own inhabitants. Further, economic growth of ancient cities offered various non-agricultural jobs that paid higher salaries as compared to those of the agriculture sector. Hence, people employed as labor shifted from the traditional agricultural sectors. Furthermore, towns and villages (including the oasis) have been aided by the government in terms of housing loans and other related types of assistant. The interaction between climatic and LULC factors have had a vital influence on ecosystem progression, as stated in previous studies [45, 46], indicating that changes in LULC have been a result of direct environmental influence of economic liberalization and globalization.

### Anthropogenic impact assessment

It can eventually summarize the main cause of urban sprawl in Al-Hasa oasis in economic factors, where changes in LULC have arisen from the response of population to the new economic situation. Hence, the urban-vegetation relationship is important as development of agricultural practices in the oasis has converted the agriculture-based nature of the oasis into a cultural landscape with components of natural heritage, causing a severe invasion of urban features into the green land cover class. These factors had a direct influence on land management. Environmental/climatic conditions could also be taken into account as the interaction between climatic and LULC factors have a significant influence on ecosystem progression, as stated by Zeng[45]. Barbier [46] indicated that economic liberalization and globalization have had a major and direct influence on changes in LULC.

Alghannam [26] proved from their study conducted in the Al-Hassa oasis that increasing vegetation cover was an effective way to cool urban areas, save energy, and improve the urban environment. Supporting that, several studies revealed that a strong and uneven urban growth increases land surface temperatures (LST) in a newly urbanized area [47,48]. Buyadi [49] also suggested that different LULC types have different LSTs, which is significantly influenced by the vegetation cover. LULC is a consequence of human activities engaged with the global environmental changes as referred by Erb [50], who also proposed that land use is a prime component of the interactions between society and nature that lead to changes in terrestrial ecosystems. Similar findings regarding LULC in the Al-Hassa oasis were also observed by other researchers [14, 18, 51]. It is possible that demographic changes caused by expansion in urbanization will eventually lead to a degradation in the fertility of lands [52]. This study found that the LULC change brought about by the population growth in the Al-Hassa oasis would have negative impacts on the climate of the area. Further, the study revealed that new expansions within the new oasis will not be able compensate the environmental equilibrium that has been lost in the old oasis.

## Conclusion

Remote sensing and GIS techniques were assessed to be useful tools to depict spatial and temporal changes in land cover at the Al-Hassa oasis. This was consistent with the findings of many other studies, which emphasize on the fact that understanding the nature of surface cover changes, besides quantifying the losses from cultivated lands, is of great importance for restoration and future rehabilitation of agriculture. The economic impact resultant from vegetation degradation hasn't been among the study scope, due to the lack of relevant data. Changes in hydrogeological condition at both areas throughout the study period, and its effect on biosystem and oasis microclimate, hasn't been included as forcing element in the oasis's demography, as well. Fairly, it was intended to highlight and quantify the urban sprawl influence on the green cover that was well observed from the obtained results. However, the following conclusions are inferred from the study:

- Change detection technique was applied in order to classify variations among different surface cover aspects, during three successive stages between 1985 and 2017 using two scenarios.
- During the first stage, significant urban sprawl (i.e., 3,200 ha) occurred on bare lands within the old oasis, while only 590 ha of the oasis’s vegetation area was occupied by urban cover.
- Unlike the first stage, the final stage revealed rapid urban development (1,270 ha by 2017) within the oasis’s vegetation region, while no urban sprawl occurred on bare lands (area of 1,900 ha, same as that in 1999–2013).
- The study provides quantitative information on the influence of urban development on the spatial changes in vegetation cover of the oasis, especially during recent decades.

## Supporting information

S1 Table Classification statistics for scenario I. (XLSX)

S2 Table Classification statistics for scenario II. (XLSX)

S3 Table Change detection analysis (images differencing) for scenario II. (XLSX)
